# Can you hear me now? The quest for better guidance on omega-3 fatty acid consumption to combat hearing loss

**DOI:** 10.6061/clinics/2016(08)01

**Published:** 2016-08

**Authors:** Ana C. Fiorini, Orozimbo A. Costa, Fulvio A. Scorza

**Affiliations:** I Pontifícia Universidade Católica de São Paulo (PUC-SP), Programa de Estudos Pós-Graduado em Fonoaudiologia, São Paulo/SP, Brazil; II Escola Paulista de Medicina/Universidade Federal de São Paulo (EPM/UNIFESP), Departamento de Fonoaudiologia, São Paulo/SP, Brazil; III Universidade de São Paulo, Faculdade de Odontologia de Bauru, Departamento de Fonoaudiologia, Bauru/SP, Brazil; IV Escola Paulista de Medicina/Universidade Federal de São Paulo (EPM/UNIFESP), Disciplina de Neurociência, São Paulo/SP, Brazil

Few things are more important to an older adult than safety and high health-related quality of life. Equally relevant is the ability of those surrounding such an adult to understand that individual’s functionality. However, achieving this reality is not simple because most people do not have experience living with older adults who have multiple, complex or even distinct health problems, including hearing loss.

Although a large proportion of adults retain good hearing throughout life, age-related hearing loss (A-RHL) is quite common among elderly individuals 1. Indeed, A-RHL is the most common sensory deficit in older adults 1,2 and occurs in response to a multifactorial process associated with insults to the auditory system that are received throughout an individual's life. These insults arise from ageing, noise damage, genetic susceptibility, otological disorders and exposure to ototoxic agents 1,2. A-RHL is characterized by reduced hearing sensitivity and decreased understanding of speech in noisy environments, as well as slowed central processing of acoustic information and impaired localization of sound sources 1,2. Epidemiologically, the hearing loss data reported in the literature are quite serious. In older adults, hearing loss is the second most common condition causing handicap, following only arthritis 3. As such, A-RHL is a major social and public health problem 1,2. Furthermore, 10% of the population has such significant hearing loss that communication may be impaired, and this rate increases to 40% in individuals older than 65 years 1,2,4. It is notable that 80% of all hearing loss cases affect the elderly population 1,2,5. Another significant issue in both developed and developing countries is the fact that A-RHL leads to adverse effects on the physical, cognitive, emotional, behavioral and social functioning of older adults, dramatically reducing the quality of life of these individuals 1,2,6. For treating A-RHL, it has been established that when hearing aids no longer provide an advantage, cochlear implantation may be effective for elderly patients, including those over eighty years old 2.

While no treatment is currently capable of restoring lost hearing, sophisticated experimental and human studies into hearing restoration have been conducted over the last decade 1,2. One goal to prevent hearing deterioration is the creation of strategies other than medical and surgical therapies that could be beneficial. Nutritional approaches, such as supplementation with omega-3 polyunsaturated fatty acids (omega-3 FAs), could have an interesting role in this context.

Omega-3 FAs, primarily eicosapentaenoic acid (EPA) and docosahexaenoic acid (DHA), are considered essential fatty acids 7-9. The designations “omega-3” or “n-3” describe long-chain fatty acids that possess a double bond located at the 3^rd^ carbon from the end of the carbon chain 7-9. The human body cannot synthesize omega-3 FAs and they must therefore be obtained from food 10-12. Thus, the advantages and nutritional benefits of a diet rich in fish and other seafood are mainly related to the high-quality protein and high concentrations of EPA and DHA that are present in these animals 13-15. The fish species with the highest levels of omega-3 FAs and lowest levels of contaminants (e.g., methylmercury, polychlorinated biphenyls and dioxins) are anchovies, Atlantic herring, salmon, trout and sardines 13,16-20; as such, these are the best choices for consumption. Early reports from Bang and Dyerberg demonstrated that Greenland Eskimos, who have diets rich in omega-3 FAs, have a low incidence of cardiovascular disease. Following these reports, a series of animal and clinical studies were designed to evaluate the positive effects promoted by omega-3 FAs in human health and disease 10,21. A number of studies have reported that omega-3 FAs are clinically beneficial and reduce triglycerides, improve cardiac health and reduce the risk of death after myocardial infarction 22-24. Additionally, omega-3 FAs impact a variety of other bodily systems and can affect the outcomes of various diseases, including metabolic and inflammatory diseases, cancer, neurological disease and mental illness 7,17,25-30. Based on these facts, several national and international guidelines have been developed with vigorous recommendations for omega-3 FA consumption by the general population for the prevention of some chronic diseases. These recommendations include the consumption of at least 250 mg/day of long-chain omega-3 FAs or at least 2 servings/week of oily fish 31. These recommendations have been made regardless of variations in the natural consumption of omega-3 FAs among different populations and cultures.

With regard to hearing loss, the importance of omega-3 FA and fish consumption on the risk of A-RHL was elegantly demonstrated by Gopinath et al. 32. They showed that dietary intervention with omega-3 FAs may be useful in preventing or delaying the development of A-RHL. These benefits may be related to the ability of polyunsaturated fatty acids to promote healthy auditory function through the maintenance of adequate vascular supply to the cochlea 32,33. Additionally, polyunsaturated fatty acids attenuate inflammatory processes 32,34, decrease blood pressure and improve vascular reactivity or endothelial function 32,35. More recently, Curhan et al. 36 conducted a prospective cohort study to evaluate independent associations between consumption of total and specific types of fish, consumption of long-chain omega-3 FAs, and self-reported hearing loss in women. These associations were examined in 65,215 women who were followed from 1991 to 2009. In brief, after 1,038,093 person-years of follow-up, 11,606 cases of incident hearing loss were reported. Based on these data, the authors demonstrated that regular fish consumption (2 or more servings of fish per week) and higher intake of long-chain omega-3 FAs are associated with a lower risk of hearing loss in women. Martínez-Veja et al. 37 used a classical mouse model of early hearing loss (C57BL/6J mice) to demonstrate that long-term omega-3 FA supplementation exerts a protective effect on cochlear metabolism and progression of hearing loss.

Hearing loss in the elderly significantly impacts everyday life. Due to the aging of populations in developed and developing countries 1,2,38, A-RHL remains a problem for all social classes that extensive public health awareness campaigns should be created to address. Although future large prospective studies exploring the links between dietary omega-3 FAs and hearing health still need to be conducted 32, the proposal of using omega-3 FA dietary interventions to prevent hearing loss in older adults is innovative, practical to apply and expected to be useful. Finally, as a deeper understanding of the emerging role of omega-3 FA consumption in preventing A-RHL is essential for the design of better strategies to treat this condition, multidisciplinary approaches in clinical and experimental translational research are crucial at this time.
